# Redox theory in progeria

**DOI:** 10.18632/aging.104211

**Published:** 2020-10-31

**Authors:** Ricardo Villa-Bellosta

**Affiliations:** 1 Fundación Instituto de Investigación Sanitaria, Fundación Jiménez Díaz (FIIS-FJD), Universidad Autónoma de Madrid, Madrid 28040, Spain

**Keywords:** aging, treatment, progeria, magnesium, glutathione

Scientific investigations, over the past centuries, into longevity and aging have led to the identification of many important hallmarks of aging and the formulation of many hypotheses [1]. For example, the rate-of-living hypothesis, first proposed in 1928, states that longevity may be regarded as a metabolic characteristic [2,3]. This hypothesis may be explained biochemically by the free radical theory of aging, in which oxygen free radicals, called reactive oxygen species (ROS), attack various macromolecules in cells, including proteins, lipids, and deoxyribonucleic acid, causing structural damage to these macromolecules and inducing functional cell senescence [2,3]. Because mitochondria are the main intracellular sources of ROS, the free radical theory was extended to the mitochondrial free radical theory of aging. Moreover, oxidative stress was defined in 1985 as “an imbalance between oxidants and antioxidants in favor of the oxidants, leading to a disruption of redox signaling and control and/or molecular damage” [2,3]. Because oxidative stress has also been observed in the absence of free radicals, the free radical theory of aging was extended, resulting in the formulation of a redox hypothesis of oxidative stress.

In recent years, several major studies have shifted the focus on oxidative stress to a more general approach focusing on redox biology. This has led to the formulation of a redox-stress theory of aging [2,3]. The cellular balance between oxidation and reduction reactions (redox state) plays a critical role in aging and is collectively determined by the reduction potentials and reducing capacities of redox couples, including glutathione (GSH)/glutathione disulfide (GSSG); the reduced and oxidized forms of nicotinamide adenine dinucleotide (NAD), NAD phosphate (NADP), and thioredoxine; and cysteine/cystine. Notably, genes associated with cytoprotective defense systems, including those encoding proteins involved in GSH production and GSH/NADPH regeneration, are regulated by nuclear factor erythroid 2-related factor 2 (NRF2), indicating that NRF2 regulates the redox state. Thus, loss of NRF2 function is key piece to many pathologic conditions, including aging and age-related diseases [4,5]. Progerin has been shown to trap NRF2 at the nuclear periphery, thus impairing its activity [4]. NRF2 sequestration by progerin results in chronic oxidative stress and contributes to HGPS aging defects, including ER stress [5], which can be reversed by the reactivation of NRF2 [4].

Although various strategies have been employed to prolong the lifespan of animals, the most effective thus far are those that augment the reducing power of cells, specifically the supply of GSH and NADPH. These pools are directly involved in the maintenance of the glutathione redox system and can be considered markers of redox status. Notably, the overexpression of genes that have a direct effect on cellular redox status, including genes encoding glutamate-cysteine ligase and glucose-6-phosphate dehydrogenase, which increase intracellular pools of GSH and NADPH, respectively, can significantly extend the lifespan of *Drosophila melanogaster* [3]. By contrast, manipulating the expression of genes with antioxidant function but without impact on redox state, such as those encoding superoxide dismutase (SOD) and catalase, has minimal effects on longevity. Notably, a new study showed that dietary magnesium supplementation improves the NADPH-coupled glutathione redox system, increasing both GSH/GSSG and NADPH/NADP ratios, reducing ROS production, and significantly extending longevity in a mouse model of progeria [6].

Hutchinson-Gilford progeria syndrome (HGPS), characterized by accelerated aging, greatly resembles the normal aging. HGPS has also been associated with mitochondrial dysfunction, reducing the availability of ATP and survival and thereby inducing vascular calcification [6,7]. HGPS has also been characterized by impaired mitochondrial membrane potential [6]. Treatment with magnesium enhanced the activity of mitochondrial proton pumps (complexes I, III, and IV), stimulated extramitochondrial NADH oxidation, and enhanced coupled mitochondrial membrane potential. These processes increased H+-coupled mitochondrial NADPH and ATP synthesis, the latter of which is necessary for cellular energy supply and survival [6]. Moreover, magnesium treatment also reduced calcification in the aortic wall [6]. The dependence of both NADPH and ATP supplies on common precursors links bioenergetic responses to diet and oxidants to the flexibility of redox systems. For example, impaired mitochondrial ATP supply stimulates glycolysis, thereby limiting the pentose phosphate pathway supply of NADPH needed to maintain GSH and thioredoxin functions, both of which are NADPH-coupled redox systems. Notably, magnesium treatment of mice with HGPS showed reductions in glycolysis and, therefore, reductions in lactate and hydrogen ions (acidosis) [6].

Magnesium deficiency is involved in aging and several diseases, including cardiovascular and metabolic diseases. Magnesium plays a critical role in more than 300 enzymes, including those involved in energy metabolism, mitochondrial function and protein/nucleic acid synthesis. Notably, treatment of HGPS mice with magnesium enhanced cellular senescence and loss of cellular replication [6].

Although aging is very often associated with magnesium inadequacy, total plasma magnesium concentration (which represents about 1% of magnesium in the body) is remarkably constant in healthy subjects throughout life and does not tend to change with aging. Although plasma magnesium concentrations do not change markedly in children with HGPS and in elderly people, magnesium supplementation may constitute a new therapeutic approach for patients with this devastating disease and may promote healthier aging in the general population. In conclusion, this new study [6] showed that the antioxidant properties of magnesium improved redox status and longevity, thus supporting the redox theory of aging [2,3].

## References

[1] López-Otín C, et al. Cell. 2013; 153:1194–217. 10.1016/j.cell.2013.05.039 23746838 PMC3836174

[2] Jones DP. Redox Biol. 2015; 5:71–79. 10.1016/j.redox.2015.03.004 25863726 PMC4392062

[3] Sohal RS, Orr WC. Free Radic Biol Med. 2012; 52:539–55. 10.1016/j.freeradbiomed.2011.10.445 22080087 PMC3267846

[4] Kubben N, et al. Cell. 2016; 165:1361–74. 10.1016/j.cell.2016.05.017 27259148 PMC4893198

[5] Hamczyk MR, et al. EMBO Mol Med. 2019; 11:e9736. 10.15252/emmm.201809736 30862662 PMC6460349

[6] Villa-Bellosta R. EMBO Mol Med. 2020; e12423:e12423. 10.15252/emmm.202012423 32875720 PMC7539193

[7] Villa-Bellosta R. Proc Natl Acad Sci USA. 2019; 116:23698–704. 10.1073/pnas.1910972116 31690656 PMC6876227

